# Association between molecular markers and resistance to bacterial blight using binary logistic analysis

**DOI:** 10.1186/s12870-024-05381-1

**Published:** 2024-07-15

**Authors:** Seyyedeh Zahra Fatemifard, Asad Masoumiasl, Rasool Rezaei, Bahman Fazeli-Nasab, Ali Salehi-Sardoei, Mansour Ghorbanpour

**Affiliations:** 1 Plant Breeding Department, Agriculture Faculty, Yasouj University, Yasouj, Iran; 2 Plant Protection Department, Agriculture Faculty, Yasouj University, Yasouj, Iran; 3 Department of Agronomy and Plant Breeding, Agriculture Institute, Research Institute of Zabol, Zabol, Iran; 4 Crop and Horticultural Science Research Department, South Kerman Agricultural and Natural Resources Research and Education Center, AREEO, Jiroft, Iran; 5 Department of Medicinal Plants, Faculty of Agriculture and Natural Resources, Arak University, Arak, 38156-8-8349 Iran

**Keywords:** Genetic diversity, ISSR, Polymorphism, *Pseudomonas syringae*, Resistant wheat cultivars

## Abstract

The most effective strategy for managing wheat bacterial blight caused by *Pseudomonas syringae* pv. *syringae* is believed to be the use of resistant cultivars. Researching the correlation between molecular markers and stress resistance can expedite the plant breeding process. The current study aims to evaluate the response of 27 bread wheat cultivars to bacterial blight disease in order to identify resistant and susceptible cultivars and to pinpoint ISSR molecular markers associated with bacterial blight resistance genes. ISSR markers are recommended for assessing a plant's disease resistance. This experiment is focused on identifying ISSR molecular markers linked to bacterial blight resistance. After applying the bacterial solution to the leaves, we performed sampling to determine the infection percentage in the leaves at different intervals (7, 14, and 18 days after spraying). In most cultivars, the average leaf infection percentage decreased 18 days after spraying on young leaves. However, in some cultivars such as Niknegad, Darab2, and Zarin, leaf infection increased in older leaves and reached up to 100% necrosis. In our study, 12 ISSR primers generated a total of 170 bands, with 156 being polymorphic. The primers F10 and F5 showed the highest polymorphism, while the F7 primer exhibited the lowest polymorphism. Cluster analysis grouped these cultivars into four categories. The resistant group included Qods, Omid, and Atrak cultivars, while the semi-resistant and susceptible groups comprised the rest of the cultivars. Through binary logistic analysis, we identified three Super oxide dismutase-related genes that contribute to plant resistance to bacterial blight. These genes were linked to the F3, F5, and F12 primers in regions I (1500 bp), T (1000 bp), and G (850 bp), respectively. We also identified seven susceptibility-associated genes. Atrak, Omid, and Qods cultivars exhibited resistance against bacterial blight, and three genes associated with this resistance were linked to the F3, F5, and F12 primers. These markers can be used for screening or transferring tolerance to other wheat cultivars in breeding programs.

## Introduction

Wheat is the primary food source for nearly two billion people, accounting for 36% of the world's population, especially in the Middle East and southwest Asia. It contributes to about 55% of the carbohydrates consumed and 20% of the world's calorie supply [1]. With the UN predicting that the world population will reach 10 billion by 2050, the significance of grains as the staple food source will increase even further. Therefore, research aimed at increasing wheat productivity will be extremely valuable [2–4].

In some parts of the world, epidemics or outbreaks of pests have resulted in the complete loss of plant production, impacting various aspects of food security and the health of ecosystems [5]. Wheat bacterial blight disease leads to reduced plant height, grain yield, and yield components, ultimately stunting plant growth. It causes gray-green to reddish-brown spots on the leaves, which can spread, causing necrosis and eventually melanosis in the entire leaf. Grain contamination plays a significant role in the spread of the disease. Therefore, transporting infected grains can lead to the spread of the disease to uninfected areas [5]. The use of antibiotics in disease management is not recommended due to the high cost and the development of resistant bacterial populations. It appears that the best way to manage this disease is by using resistant wheat cultivars [6]. The focus of recent research in plant pathology is to find ways to control plant diseases with minimal environmental impact. The most promising strategy is the production of disease-resistant plants through traditional plant breeding or genetic engineering techniques. Additionally, the use of agronomic measures to suppress the disease, gene silencing techniques, non-toxic additives, and biological agents that counteract microorganisms are well-known methods to achieve this goal [7–10].

Bacterial blight (BB) is a widespread disease that significantly affects wheat crops, causing substantial yield losses and decreased productivity. This disease is triggered by the bacterium *Xanthomonas oryzae* and presents a significant challenge in achieving high and consistent rice grain yields. Therefore, it is crucial to develop wheat varieties that are resistant to BB to minimize its impact. Multiple resistance genes have been identified in wheat, such as xa5, which encodes a unique form of disease resistance. The genetic mechanisms involved in bacterial blight resistance in wheat involve the interaction between the host and the pathogen. This interaction is mediated by resistance (R) genes, sensitivity (S) genes, SSP genes, and QRLs. Breeding strategies for bacterial leaf blight resistance in rice involve utilizing classical R genes and QTLs. Additionally, Marker-Assisted Selection (MAS) is employed to introduce these genes into wheat varieties. Continuous and diligent efforts are necessary to identify sources of novel genes/QTLs capable of overcoming newly emerging pathogen races and providing long-term field resistance. Molecular markers linked to these R genes and QTLs are available, enabling MAS to develop resistant wheat varieties. It is important to note that CRISPR/Cas9-targeted mutagenesis has been used to confer resistance to *X. oryzae* in rice, and this approach can also be applicable in wheat. Understanding the history and significance of bacterial blight disease in wheat, along with the genetic mechanisms and breeding strategies, is essential for the development of new resistant wheat varieties [11, 12].

The relationship between markers and genotypes is crucial for identifying the genetic basis of resistance to bacterial blight. Markers can be used to identify specific genes or regions associated with resistance. This information can then be used to develop resistant varieties. The effectiveness of resistance genes can be influenced by variations in the *X. oryzae* bacterium. Therefore, it is important to assess resistance under different pathotypes and environmental conditions. By combining the xa5, xa13, and Xa21 genes, resistance to the disease can be improved. Continuous efforts are needed to find sources for new genes/QTLs to overcome new emerging races of the pathogen and achieve long-term resistance in the field. Molecular markers associated with R genes and QTLs are currently available, which can be used for marker-assisted selection (MAS) in the development of resistant wheat varieties [13, 14].

The genetic diversity of germplasm is crucial for identifying and transferring genes to improve crops. DNA molecular markers, such as ISSR (Inter-Simple Sequence Repeat) markers, are used to assess genetic diversity and locate resistance genes against different types of stresses [15]. ISSR markers utilize microsatellite or simple sequence repeat regions of the genome to create DNA fingerprints. These markers target di-, tri-, tetra-, or pentanucleotide repeat regions within the genome and are more reproducible than RAPD markers. ISSR analysis can be used for genetic diversity assessment, fingerprinting, and studying phylogenetic relationships within and between plant species. Additionally, there are also IRAP (Inter-Retrotransposon Amplified Polymorphism) markers, which use primers designed from the long terminal repeat regions of retrotransposons to generate DNA fingerprints [16–19].

In a previous study [20], 115 wheat genotypes were evaluated for resistance to powdery mildew disease. The genotypes were genotyped using ISSR, iBPS, and IRAP markers. The ISSR and iBPS primers showed the highest and lowest percentages of polymorphism, with 100% and 50% respectively. The PR50-60 allele had the highest correlation with the traits evaluated in this study. The correlations between markers and the powdery mildew disease trait revealed only one allele associated with the disease. Additionally, five genomic fragments, ISSR10, 826, UBC840, 12,826, and LBMB, were identified using stepwise regression of alleles related to powdery mildew disease resistance in barley [21]. In a study on mutant barley cultivars, genotyped using ISSR and RAPD markers, it was stated [22] that all the studied cultivars were resistant to rust powdery mildew. Another study [23] utilized nine ISSR primers to identify genetic diversity associated with Fusarium resistance in chickpea, producing a total of 61 bands, out of which 44 (72.1%) were polymorphic. Using binary logistic regression analysis, three disease-related markers, including UBC864400bp, UBC811250bp, and UBC811650bp, were found to be associated with disease resistance. The use of markers and morphological traits in breeding programs can help identify suitable parents for producing mapping populations and hybrid cultivars. This can be particularly useful when no other genetic information is available [24]. Molecular markers have been used to select wheat varieties resistant to *Puccinia recondite* f. sp. *tritici*, and they can be a valuable tool alongside classical breeding methods [24]. In our current study, we are evaluating the response of 27 bread wheat cultivars to bacterial blight disease to identify both resistant and susceptible wheat cultivars, as well as ISSR molecular markers associated with bacterial blight resistance genes.

## Materials and methods

### Prepared bacteria and wheat cultivars

For bacterial evaluation, a 16-line culture was performed using a bacterial suspension prepared from Plant Protection Laboratory of Yasouj University. Then, to confirm the cultured specimen, gram, catalase and oxidase tests were performed [25]. Twenty-seven Iranian native wheat cultivars were prepared from Seed and Plant Improvement Institute (Karaj, Iran) (Table 1).

**Table 1:** The list of the studied Iranian native wheat cultivars

Row	Cultivar Name	Origin	Family tree	Growth Type	Resistance to disease
**1**	Azadi	Iran (Karaj-SPII)	20*1–32-15,409*Mexp	Autumn	Relatively resistant to yellow rust and semi-resistant to semi-sensitive to black and brown rust
**2**	Alamut	Iran (Karaj-SPII)	Kavz/Ti71/3/Maya''s''//Bb/Inia/4/Kj2/5/Anza/3/Pi/Ndr//Hys	Autumn	Resistant to yellow rust—sensitive to brown rust
**3**	Atrak	Mexico (CIMMYT)	Kauz”s”	Spring	Resistant to yellow and brown rust diseases and semi-tolerant to Fusarium spike
**4**	Omid	Iran (saveh)	Native (with number 1–29-11,085)	Spring	Sensitive to yellow, black and brown rust and hidden and obvious black and wind
**5**	Inia ‬‬‬‬‬‬‬‬‬	Mexico (CIMMYT)	LR64/SN64	BSA	Relatively resistant to yellow rust and semi-resistant to semi-sensitive to black and brown rust
**6**	Bezostaya	Russia	-	Autumn	Resistant to yellow rust and susceptible to brown and black rust
**7**	Boolani	Iran(SPII)	-		
**8**	Tajan	Mexico (CIMMYT)	Bow”s”/Nkt”s”(CM67428-GM-LR-5 M-3R-LB-Y)	Spring	relative resistance to yellow and brown rust and tolerant to spike Fusarium and tolerant to sprouting on the spike
**9**	Chamran	Mexico (CIMMYT)	Attila, (CM85836-50Y-OM-OY-3 M-OY)	Spring	Resistant to yellow and brown rust
**10**	Darab2	Mexico (CIMMYT)	Maya"s"/Nac	Spring	Relative resistance to yellow and brown rust, sensitive to septoria
**11**	Roshan	Iran(Isfahan)	Native	BSA	Susceptible to brown, yellow, black and hidden rust
**12**	Zarin	Mexico (CIMMYT) and Syria (ICARDA)	PK15841	BSA	Resistant to yellow rust and sensitive to brown rust, resistant to hidden black
**13**	Sabalan	Iran(SPII)	*1–32-4382(908*FnA12)	Autumn	Relatively resistant to hidden black and yellow rust
**14**	Sorkh Tokhm	Iran (Zabol)	Native	Spring	Yellow rust tolerant
**15**	Sardari	Iran (Kurdistan)	Native	Autumn	Sensitive to hidden yellow and black rust
**16**	Sholeh	Mexico (CIMMYT)	Bow”s”/Nkt”s”(CM67428-GM-LR-5 M-3R-LB-Y)	Spring	relative resistance to yellow and brown rust and tolerant to spike Fusarium and tolerant to sprouting on the spike
**17**	Tabasi	USA(Oregon) and Turkey (CIMMYT)	Kirkpinar*2–66/112–63(79C)	BSA	Semi sensitive to yellow rust and resistant to brown rust
**18**	Falat	Iran (Karaj-SPII)	-	Spring	Sensitive to yellow rust
**19**	Qods	Iran (Karaj-SPII)	Rsh/5/Wt/4/Nor10/K54*2//Fn/3/Ptr/6/Omid//Kal/Bb	BSA	Resistant to various fungal diseases and susceptible to yellow rust
**20**	Karchia	Iran (Karaj-SPII)	–	–	Salt-tolerant
**21**	Kavir	Iran (Zabol-SPII)	Stm/3/Kal//V534/Jit716	Spring	Resistant to salinity and tolerant to drought at the end of the season and wind
**22**	Golestan	Mexico (CIMMYT)	Alondra”s”	Spring	Semi-sensitive to yellow, black and brown rusts
**23**	Mahouti	Iran (Karaj-SPII)	-	Autumn	Salt-tolerant
**24**	Mahdavi	Syria (ICARDA)	Ti/Pch/5/Mt48/3/Wt*//Nar59/Tota63/4/Mus	BSA	Resistant to yellow rust
**25**	Mihan	Iran	B k t /90-Zhong87	Autumn	resistant to yellow rust and semi-resistant to semi-sensitive to black and brown rust
**26**	Niknegad	Syria (ICARDA)	F13471/Crow”s”	Spring	Resistant to yellow rust disease

Seed and Plant Improvement Institute (SPII), *BSA* Between spring and autumn

### Experimental conditions

Planting was done in three replications with five wheat seeds per pot. The cultivated plants were maintained under controlled conditions of humidity, temperature (25°C), and photoperiod (8 h dark and 16 h light). Inoculation with *Pseudomonas syringae* pv. *syringae* was performed at the 4–6 leaf stages and then sampling was carried out at different times (1, 3, and 5 days after spraying). The ratio of contaminated leaf area to the total leaf area was measured with Image J software and after calculating the leaf infection percentage, the cultivars were grouped via the Milus and Chalkley [26] method.

### DNA extraction and electrophoresis

For molecular evaluation, DNA was extracted from flag leaf samples using the method described by Dellaporta et al. [27]. Table 2 lists the sequence and annealing temperature of the 12 ISSR primers and Table 3 lists the amplification reaction performed according to the temperature cycle. The relationship between bacterial blight disease in wheat and ISSR markers lies in the use of ISSR markers to assess the genetic diversity of wheat cultivars in terms of their resistance to bacterial blight. ISSR markers are particularly effective in categorizing plants based on disease resistance, and their advantage lies in not requiring DNA sequencing data beforehand. This makes them a rapid and straightforward method for identifying resistant cultivars. However, the efficiency of ISSR markers can vary depending on the primer utilized. For instance, primers F7, F8, and F10 have shown to yield the highest percentage of polymorphic bands, while primer F9 yields the lowest [28]. In order to analyze the molecular data, the PCR products were subjected to gel electrophoresis and the presence or absence of bands were scored as zero and one, and the preliminary data matrix was formed. NTSYS version 2.02 software was utilized to study the genetic relationships between the studied genotypes, grouping them, and also performing a principal component analysis. SPSS Statistics 22 software was used for binary logistic regression analyses.

**Table 2:** Sequence and annealing temperature of the primers

Primer	Annealing temperature (°C)	Sequence (3’-5’)
F1	63.6	ACC ACC ACC ACC ACC ACC G
F2	56.3	ACA CAC ACA CAC ACA CCG
F3	52.4	CTC TCT CTC TCT CTC TG
F4	43.2	GAT AGA TAG ACA GAC A
F5	36	GGG TGG GGT G
F6	52.6	GTG TGT GTG TGT GTG TYA
F7	56.3	CTC TCT CTC TCT CTC TGC
F8	43.5	CTC TCT CTC TCT CTA
F9	38	CAG CAG CAG GC
F10	43.7	GTG TGT GTG TGT CC
F11	40.8	CAC ACA CAC ACA AC
F12	40.8	CAC ACA CAC ACA GT

**Table 3:**
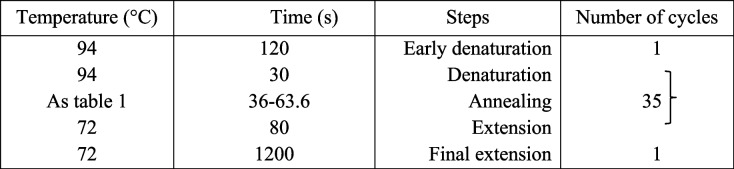
PCR program for genomic DNA replication using ISSR markers

## Results

**Fig. 1:**
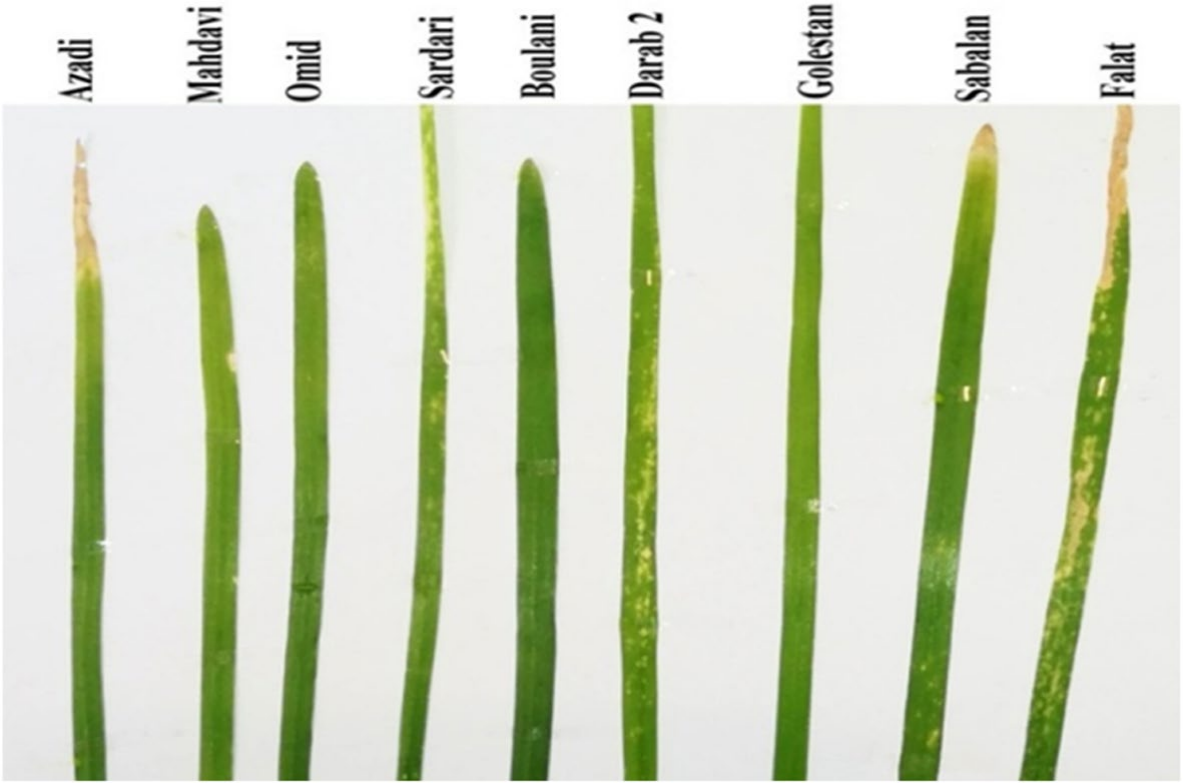
Leaf contamination symptoms of some cultivars 14 days after leaf spraying

**Fig. 2:**
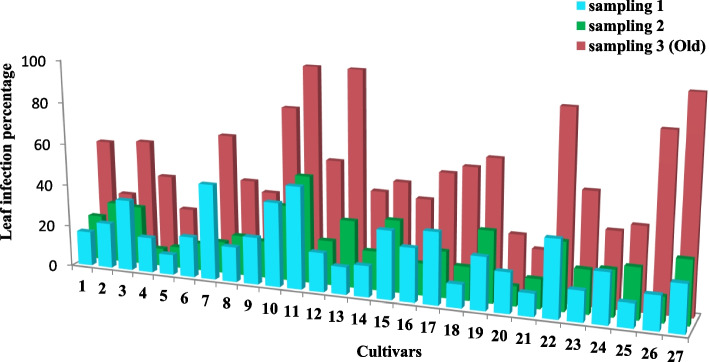
Leaf infection percentage at three harvesting (sampling) of old leaves of the studied wheat cultivars. The numbers are: 1 (Azadi), 2 (Alamut), 3 (Alvand), 4 (Atrak), 5 (Omid), 6 (Inia), 7 (Bezostaya), 8 (Boolani), 9 (Tajan), 10 (Chamran), 11 (Darab2), 12 (Roshan), 13 (Zarrin), 14 (Sabalan), 15 (Sorkh Tokhm), 16 (Sardari), 17 (Sholeh), 18 (Tabasi), 19 (Falat), 20 (Qods), 21 (Karchia), 22 (Kavir), 23 (Golestan), 24 (Mahouti), 25 (Mahdavi), 26 (Mihan), 27 (Niknegad), respectively ‬

**Fig. 3:**
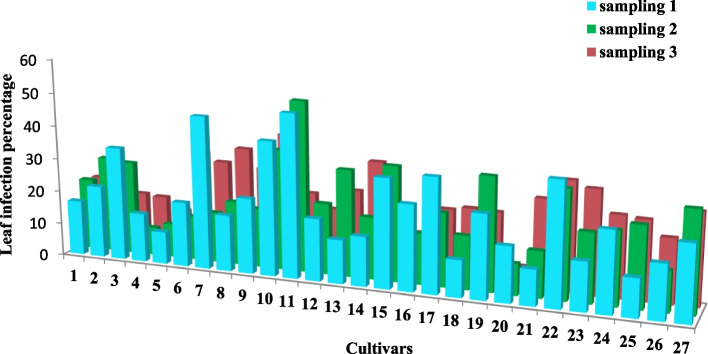
Leaf infection percentage at three harvesting (sampling) of young leaves of the studied wheat cultivars. The numbers are: 1 (Azadi), 2 (Alamut), 3 (Alvand), 4 (Atrak), 5 (Omid), 6 (Inia), 7 (Bezostaya), 8 (Boolani), 9 (Tajan), 10 (Chamran), 11 (Darab2), 12 (Roshan), 13 (Zarrin), 14 (Sabalan), 15 (Sorkh Tokhm), 16 (Sardari), 17 (Sholeh), 18 (Tabasi), 19 (Falat), 20 (Qods), 21 (Karchia), 22 (Kavir), 23 (Golestan), 24 (Mahouti), 25 (Mahdavi), 26 (Mihan), 27 (Niknegad), respectively

The symptoms of the susceptible cultivars included dead tissue strips on leaves (Fig. 1). Figures 2 and 3 depict the leaf infection percentage at three harvesting times in old and young leaves of the studied wheat cultivars. The average leaf infection at the third sampling time in young leaves decreased in most cultivars, yet increased in old leaves.

The mean comparison of infection percentage (Table 4) showed that Atrak, Omid, and Qods cultivars were resistant against bacterial blight. Although Mahdavi, Sardari, Roshan, Azadi, Karchia, Tabasi, and Mihan cultivars were categorized in the same group, they did not differ significantly from the sensitive cultivars.

**Table 4:** The mean comparison of infection percentage of three harvesting times in the studied cultivars

Cultivar	Infection percentage	Cultivar	Infection percentage	Cultivar	Infection percentage
Darab2	40.668^a^	Chamran	38.500^ab^	Kavir	33.922^a−c^
Bezostaya	30.016^b−d^	Sorkh Tokhm	27.790^c−e^	Falat	26.877^c−f^
Niknegad	26.598^c−f^	Alvand	26.356^c−g^	Sholeh	25.841^e−i^
Mahooti	23.411^d−h^	Boolani	23.201^d−h^	Zarrin	23.041^d−h^
Tajan	22.891^d−h^	Sabalan	22.207^d−h^	Golestan	22.043^d−h^
Alamut	21.950^d−h^	Inia ‬‬‬‬‬‬‬‬‬	21.331^d−h^	Mahdavi	19.729^e−i^
Azadi	19.596^e−i^	Roshan	19.240^e−i^	Sardari	18.069^f−i^
Karchia	17.098^g−i^	Tabasi	16.339^g−i^	Mihan	15.794^g−i^
Atrak	12.558^hi^	Omid	10.873^i^	Qods	10.787^i^

In each column that means have at least one same letter (a-i) are not statistically significantly different at the 5% level

**Fig. 4:**
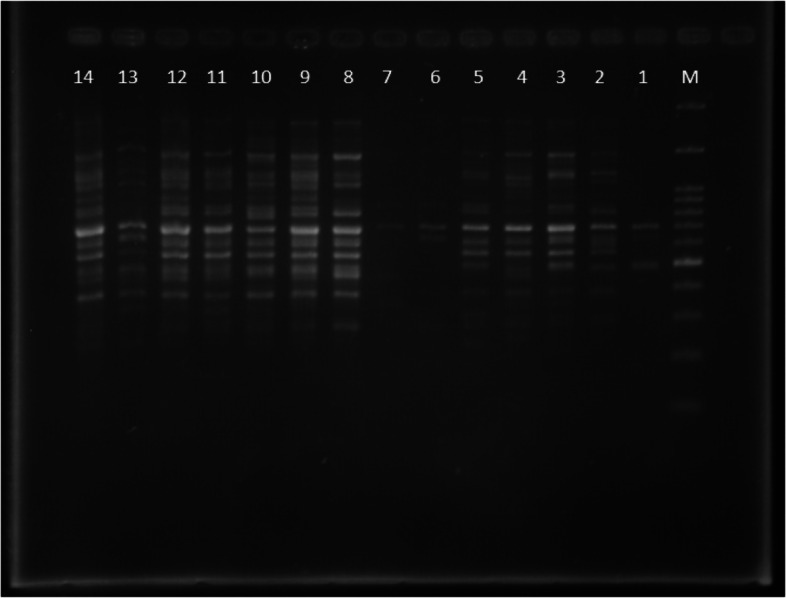
ISSR band pattern produced by F10 primer (From right to left, respectively, M is the size marker and the number 1 to 14 is the same as the numbers show genotype number as Table 1)

**Fig. 5:**
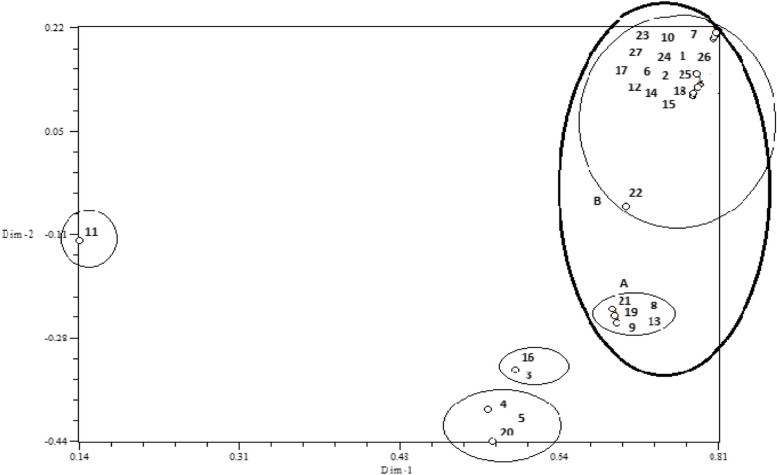
A two-dimensional graph of the principal component analysis of the studied wheat cultivars. The numbers are: 1 (Azadi), 2 (Alamut), 3 (Alvand), 4 (Atrak), 5 (Omid), 6 (Inia), 7 (Bezostaya), 8 (Boolani), 9 (Tajan), 10 (Chamran), 11 (Darab2), 12 (Roshan),13 (Zarrin), 14 (Sabalan), 15 (Sorkh Tokhm), 16 (Sardari), 17 (Sholeh), 18 (Tabasi), 19 (Falat), 20 (Qods), 21 (Karchia), 22 (Kavir), 23 (Golestan), 24 (Mahouti), 25 (Mahdavi), 26 (Mihan), 27 (Niknegad), respectively

According to the molecular analysis, 12 primers produced (Fig. 4) a total of 170 bands, of which 156 were polymorphic. Therefore, the percentage of polymorphism for all primers was 92.65%. The highest number of amplified fragments belonged to the F5 and F9 primers, with 20 fragments and the lowest number to the F8 primer with 6 fragments. Figure 5 shows the two-dimensional graph of the principal component analysis of the studied wheat cultivars. The best number of groups was considered to be four, using T^2^ Hoteling and CCC tests. The first group included Darab2; the second group consisted of Qods, Omid, and Atrak cultivars; the third group comprised Sardari, and Arvand cultivars; the fourth group consisted of two subgroups: A, including Falat, Zarrin, Karchia, Tajan, and Boolani cultivars and B, including Kavir, Tabasi, Sorkh Tokhm, Sabalan, Sholeh, Roshan, Inia, Alamut, Niknegad, Mihan, Mahdavi, Mahouti, Golestan, Chamran, Bezostaya, and Azadi cultivars.

According to molecular clustering, Sardari and Arvand cultivars were placed into the resistant cluster group. Semi-susceptible cultivars are also identified due to symptoms and molecular clustering. In this study, binary logistic regression analysis was performed where biochemical traits were considered as independent variables and the gene loci of F10 and F5 primers, which showed the most diversity, were considered as the dependent variable. The results revealed that some biochemical traits are associated with the different loci (Table 5). At F5 primer, only the F_5_-G locus with a B coefficient of 17.224 at the third post-stress harvest showed a significant relationship with SOD at the 5% probability level. Moreover, the F_12_-R locus indicated the highest correlation with SOD at 5% probability level with a B coefficient of 23.913 at the third harvest after stress. The F_3_-I locus was correlated with SOD with a B coefficient of 44.932. Given that the F_5_-G, F_12_-R, and F_3_-I loci were highly correlated with SOD, these loci might be correlated with resistance genes of wheat against bacterial blight disease. Furthermore, according to Table 5, the correlation obtained from binary logistic analysis based on disease susceptibility and considering foliar contamination at the first harvest time, F_2_-H locus (600 bp) with B coefficient of 0.171 at the third harvest time, F_10_-D (510 bp), F_10_-E (590 bp), F_11_-K (1400 bp), F_12_-B (310 bp), (1300 bp) F_12_-N, and F_12_-M (900 bp) loci with B coefficients of 0.189, 0.010, 0.156, 0.183, 0.35, and 0.187, respectively, were correlated with susceptibility of wheat to bacterial blight disease.

**Table 5:** B coefficients in binary logistic analysis with F1-F12 primers between disease resistance statuses in studied traits

Primer area (bp) Trait	2_H_ (600)	3_M_ (3100)	3_I_ (1500)	5_G_ (250)	10_D_ (510)	10_E_ (590)	11_K_ (1400)	12_B_ (310)	12_D_ (380)	12_M_ (900)	12_N_ (1300)	12_R_ (1000)
SOD (Time 1)	-532.594	-8.305	2.400	17.248^*^	-10.262	-4.948	1.556	68.020	68.020	10.913	-.683	0.439
SOD (Time 2)	-809.502	12.163	44.392^*^	-14.939	5.534	-2.916	0.486	-89.002	-89.002	-13.356	-13.938	-25.241
SOD (Time 3)	745.113	-2.680	-40.524	4.980	-20.262	-0.905	-8.807	-47.698	-47.698	-3.793	5.995	23.913^*^
POD (Time 1)	23.192	-2.109	-5.110	-5.130	0.934	-4.455	-7.207	-54.917	-54.917	-8.344	-2.394	2.585
POD (Time 2)	-176.417	-4.707	8.171	4.050	-6.267	2.089	3.981	83.735	83.735	9.108	-0.513	-3.910
POD (Time 3)	-618.069	-4.385	-11.554	-3.644	-13.300	-6.625	-4.264	-51.820	-51.820	-2.226	-3.525	2.307
Pr (Time 1)	-14.812	0.851	0.504	-0.816	-0.483	1.236	0.574	-8.207	-8.207	0.405	0.790	0.711
Pr (Time 2)	28.966	-.033	0.871	0.177	0.305	0.204	-0.944	3.353	3.353	-.370	-0.246	-0.118
Pr (Time 3)	-28.088	-0.777	-2.612	0.771	-2.140	-0.491	-0.550	8.095	8.095	1.653	0.894	-0.445
Leaf infection (Time 1)	0.171^*^	0.018	-0.003	0.014	0.018	0/054	-0.039	0.013	0.013	-0.014	0.052	0.005
Leaf infection (Time 2)	-0.208	-0.234	0.032	-0.096	-0.023	-0.131	0.101	0.024	0.024	-0.004	-0.095	-0.037
Young leaf infection (Time 3)	0.061	0.398	0.070	-0.068	0.189^*^	0.301^*^	0.156*	0.183^*^	0.183^*^	0.135^*^	0.187^*^	0.074
Old leaf infection (Time 3)	0.038	-0.043	-0.033	0.024	-0.033	-0.018	-0.019	-0.032	-0.032	0.000	0.015	0.000

*SOD* Superoxide dismutase, *POD* Proxidase, *Pr* Protein

*Correlation is significant at the 0.05 level

## Discussion

In the molecular diversity section, a total of 10 primers were used, resulting in 170 evaluable sites. Out of these sites, 156 polymorphic bands were observed. Cluster analysis based on Jaccard genetic coefficients produced a dendrogram which effectively separated different groups and categorized the genotypes into 4 main clusters. The Mahouti and Mahdavi genotypes showed the lowest genetic distance, while the Darab2 and Qods genotypes exhibited the highest genetic distance from Azadi. These findings suggest that the ISSR marker linked to the bacterial blight resistance gene in wheat can be used by breeders to rapidly and efficiently screen genotypes for this gene. Additionally, the marker contributes to the completion of the wheat genetic map. Among the primers used, F5 and F10 were identified as the most suitable for further studies due to their high polymorphism percentage and high PIC.

The symptoms of susceptible cultivars were similar to those observed in other studies [6, 28]. Niknegad, Darab2, and Zarrin cultivars showed 100% tissue mortality. This could confirm if the environmental conditions were appropriate, and the disease could cause significant loss and decrease crop yield [29]. Moreover, Darab2, Chamran, and Kavir cultivars were placed in the highly susceptible group [26]. The pathogen-host interaction is most likely an interaction between the pathogenic Avr protein and the host recognition complex, which results in signaling and a defensive response in the host. One of the primary defense responses is the hypersensitivity response, a type of programmed cell death that occurs at the site of the pathogenic intrusion, including viruses [30]. This process also occurs in other pathogens, including bacteria [7–10]. The results observed in this study showed a kind of dead tissue, which may be due to the type of reaction.

Based on previous research [6], Omid and Golestan cultivars were identified as resistant and susceptible, respectively, to bacterial blight disease. The second group was classified as resistant cultivars, while the third group was classified as semi-susceptible cultivars and the fourth group as susceptible cultivars. A study on the resistance of commercial wheat cultivars to Pss revealed that after 40 days, Roshan, Omid, Qods, and Gaspart wheat cultivars, as well as barley cultivars, showed reduced symptoms, leaving only a small amount of dead tissue [6]. Another study [31] reported that Falat, TR80, C73-5, and Arvand mutant cultivars were immunized, and Arvand, Atrak, Sorkh Tokhm, Niknegad, M-73–18, and Fr319 were resistant cultivars. However, in the current study and in the previous research [6], Sorkh Tokhm and Niknegad cultivars were found to be susceptible. The researchers also indicated that Atrak, Falat, and Arvand cultivars are susceptible, while Atrak was categorized, along with Omid, as a disease-resistant cultivar.

Differences between individuals are due to variations in their DNA sequences, which are also passed on to their offspring. Even differences in how individuals are affected by environmental conditions can be traced back to differences in DNA sequences. These differences can be used as genetic markers [32–36], indicating a correlation and linkage with the genes expressing these traits. The impact coefficient B was used to examine the relationship between the markers and biochemical traits. A positive and large B indicated the presence of the band, while a negative B indicated the absence of the band. Identifying molecular markers linked to genes that control traits is a valuable method for identifying resistant cultivars. ISSR intergenomic microsatellite markers are a type of molecular markers that rely on amplifying short DNA fragments within simple repetitive sequences [19, 35, 37, 38].

The internal fragments within indigenous Iranian wheat populations display a high degree of genetic diversity, even among closely related genotypes, due to the lack of functional constraints in these genomic regions. Understanding the genetic distance between individuals and populations, as well as their relationships, helps in selecting suitable populations for genetic mapping and gene localization. The significant level of polymorphism observed in these populations suggests that molecular markers such as ISSR are effective tools for studying their germplasm [18, 19, 32, 35, 39, 40]. This level of polymorphism not only demonstrates the usefulness of these markers in studying wheat germplasm but also indicates the broad geographical distribution and diversity of genotypes of this plant in Iran. The research findings underscore the necessity of genetic diversity among the native populations of Iranian wheat for carrying out breeding programs aimed at increasing pest and disease resistance, yield, quality, and adaptability. Additionally, the ISSR-PCR method, in conjunction with statistical multivariate methods like cluster analysis, shows considerable potential for examining kinship relationships, evolution, genetic diversity, and geographic distribution of plants.

The relationship between Inter Simple Sequence Repeat (ISSR) markers and resistance to bacterial blight was examined using binary logistic analysis. Genetic diversity in various genotypes was assessed using ISSR markers, and the results were promising. In a specific study, ISSR primers produced different numbers of DNA fragments with varying levels of polymorphism, indicating their potential in genetic analysis. Additionally, a combined analysis of RAPD and ISSR markers was used to evaluate genetic diversity and specific markers for different accessions were discovered. These findings highlight the usefulness of ISSR markers in genetic studies of bacterial blight resistance, increasing the reliability of molecular markers for such analyses [41, 42].

In plant breeding, one important factor in selecting parents for crossbreeding and creating diverse cultivars is the genetic distance between breeding materials. Cluster decomposition is used to form uniform groups. By analyzing genetic markers and creating a dendrogram, the genetic position of cultivars can be determined, making it more reliable to select parents for future breeding programs [43–49]. Based on the results of this study, it is recommended to replicate this experiment over the years. It's important to repeat the experiment in different locations using the investigated genotypes and to examine the correlation of the combined data with genetic markers in order to draw conclusions.

The study of the association between Inter Simple Sequence Repeat (ISSR) markers and resistance to bacterial blight in wheat is an important research area. ISSR markers have been utilized to explore the genetic diversity in wheat germplasm concerning resistance to bacterial blight. These markers play a crucial role in identifying resistant genes and gaining insight into the genetic basis of resistance. Marker Assisted Selection (MAS) is a valuable plant breeding technique that uses molecular markers like ISSR to selectively breed for resistance to specific diseases [50]. This approach helps in identifying and characterizing sources of resistance, which is essential for developing resistant cultivars. The use of ISSR markers, as well as other DNA markers like STS, RAPD, and SSR, has been instrumental in studying rust resistance genes and leaf rust resistance in wheat germplasm [51]. In summary, ISSR markers are essential tools in molecular breeding programs aimed at improving disease resistance in wheat varieties. Their ability to assess genetic diversity, identify resistant genes, and facilitate marker-assisted selection significantly contributes to the development of disease-resistant wheat cultivars [50, 51].

## Conclusion

This study revealed that Atrak, Omid, and Qods cultivars were resistant cultivars against bacterial blight and three genes associated with bacterial blight resistance of wheat were identified as belonging to the F_3_, F_5_, and F_12_ primers located in I (1500 bp), G (850 bp), and T (1000 bp) regions, respectively. There were also seven genes including F2-H (600 bp), F10-D (510 bp), F10-E (590 bp), F11-K (1400 bp), F12-B (310 bp), F12-N (1300 bp), and F12-M (900 bp) that associated with the disease susceptibility. The grouping of resistant cultivars in the molecular study was similar to that of the leaf symptoms and there was an insignificant difference between the susceptible cultivars.

## Data Availability

The raw data of this article will be made available by corresponding authors according to the personal requests.

## Abbreviations

BB: Bacterial blight
MAS: Marker-assisted selection
QRLs: Quantitative resistance loci
SSP: Small secreted protein
